# Upper tract access in patients with urinary diversions

**DOI:** 10.3389/fruro.2024.1392139

**Published:** 2024-06-25

**Authors:** Jonathan Modai, Yasin Bhanji, Parth M. Patel, Kymora Scotland, Matthew D. Dunn

**Affiliations:** Department of Urology, University of California, Los Angeles, Los Angeles, CA, United States

**Keywords:** urinary diversions, ureteroscopy, laser lithotripsy, percutaneous nephrolithotomy, endourology

## Abstract

Urinary tract diversions are a common reconstructive solution for patients with malignant, anatomic or functional pathologies of the lower urinary tract. Although urinary diversions often represent the patient’s best alternative, they are not devoid of complications. Some of these complications, mainly anastomotic strictures and kidney stones, can be managed endoscopically, although upper tract access of such patients is not straightforward, requiring a fundamental understanding of the many types of diversions. In this article we will review the inherent difficulties of accessing the upper tract of patient with different diversions, the different approaches to the upper tract of diverted patients, and the equipment and techniques that can help facilitate upper tract access in diverted patients.

## Introduction

First described by Simon in 1852 (1), urinary diversions offer a functional solution to patients with malignant, functional and congenital pathologies of the lower urinary tract. While the original design achieved unsatisfactory results, the design improved with time, and today modern urologists can offer a wide range of optional diversions, the vast majority of which use a gastrointestinal (GI) graft to reconstruct the lower urinary tract. Diversions are generally categorized into 3 subgroups which include conduits, continent cutaneous reservoirs and orthotopic neobladder, with each subgroup having unique advantages and shortcomings. The diversions differ in their construction and day to day function which allows each patient to be fitted with a diversion best suited for his\her lifestyle and capabilities, while taking into account chronic afflictions and past medical and surgical history.

## So why is endoscopic access to the kidney important in diverted patients?

The first descriptions on endoscopic access to the upper urinary tract were reported in 1975 when Goodman and Lyon reported on their experience using a pediatric cystoscope to survey an adult’s ureter (2, 3). Since then, advancements in optics and camera design have allowed for the development of flexible ureteroscopes, which became smaller and more maneuverable over the years. This allows us to treat afflictions of the upper tract urinary system, such as kidney stone disease, upper tract urothelial carcinoma and upper tract strictures, in a minimally invasive way, becoming one of the most utilized surgical treatments in the field of urology. The utilization of endoscopic treatments in diverted patients is especially important for several reasons. First, diverted patients have undergone abdominal surgery, both for their original pathology and for the diversion construction, making any further open\robotic surgical intervention technically more challenging with a higher risk for complications. Moreover, diverted patients have a higher risk to require repeated upper tract surgical interventions, making minimally invasive treatment options very alluring. This is due to their risk of developing uretero-enteric anastomotic strictures, estimated to be between 3–10% on long term follow up (3). Furthermore, ileal conduits, one of the most utilized diversions, carry an increased risk to form kidney stones, with a rate reported to be as high as 15.3% (4), almost twice the rate reported in the general population (5). The propensity of other urinary diversion to form kidney stone is less obvious, with most reports agreeing kidney stone formation rate are similar to the general population (6–8).

However, endoscopic upper tract access and manipulation can prove challenging in diverted patients owing to their unique anatomy. This translates to a reported endoscopic upper tract access success rate of between 33–90% depending on the type of diversion and the indication for which endoscopic upper tract access was attempted (9). It also explains the increase in post operative complication rate reported to be as high as 33% (10, 11).

Understanding the unique challenges of endoscopic upper tract access can assist urologists who plan to tackle them.

## What challenges can we expect and how do we deal with them?

Before attempting endoscopic upper tract access in diverted patients, it is important to do a urine culture, as more often than not, the lower urinary tract of these patients is colonized by bacteria due to the incorporation of gastrointestinal graft into the urinary system and the common use of clean intermittent catheterization among them. Identifying the colonizing bacteria and treating the patients several days prior to the procedure will lower the risk of post-surgical infectious complications.

The first challenge with any diverted patient is navigating the reconstructed diversion. Conduits are relatively straight forward and can usually be scoped without much difficulty with a rigid or flexible cystoscope. Nevertheless, some conduits can be challenging, especially older conduits as they tend to elongate with time and become convoluted. Good fluid flow and slow manipulation are key. When the way forward becomes uncertain, slowly pulling the cystoscope out can usually allow the surgeon to identify the conduits lumen. Continent cutaneous reservoirs and orthotopic neobladder are more challenging to navigate. While gaining access to orthotopic neobladder through the urethra is straightforward, accessing continent cutaneous reservoirs faces the same challenges as navigating a conduit. Once inside the reservoirs themselves, navigation with a flexible cystoscope is extremely difficult due to the large volume and irregular shape of the reservoir, and the flimsy nature of the cystoscope. Continent cutaneous reservoirs are even more challenging owing to the convoluted access through the stoma limiting any further manipulation of the cystoscope once it is inside the reservoir. Rigid cystoscopes and nephroscopes are easier to handle inside most reservoirs but can rarely reach all the areas within them. There are several techniques that can help mitigate these challenges. In cases where the stoma diameter allows, placing some type of an access sheath through the stoma can help, making reservoir access effortless and flexible cystoscopes manipulation easier. Another technique that helps navigating reservoirs, is keeping them relatively empty, which allows flexible and rigid cystoscope, and nephroscopes to reach remote areas that move away when full. One caveat to this maneuver is that empty reservoirs have folds which can hide the ureteral orifice (UO).

The second challenge to accessing the upper tract in diverted patients is UO identification, which can be difficult for several reasons. First, the UOs position varies between different types of diversions, and even between patients with the same type of diversion. Thus, it requires the surgeon to have a solid understanding of the particular reservoir anatomy so as to know where to find the UO. Moreover, the UO entry point can at times come into the diversion in an awkward angle, making identification of the UO difficult. Furthermore, UO identification can be challenging due to the fact that the diversions, being constructed from a GI graft, tend to have many folds which might obscure the UOs within them. UO identification can be challenging in all diversions, but the degree of difficulty usually depends on the diversion type, with conduits being the easiest owing to their low volume and simple architecture, while right colon reservoirs and Indiana pouches being almost impossible to access in a retrograde fashion due to the relative positions of the afferent and efferent limbs. As mentioned before, having access to both flexible and rigid cystoscopes and nephroscopes, utilizing a sheath through the stoma when relevant and possible, and scoping the reservoirs at different volumes can help identify the UOs. Administering fluoroscein, indigo carmine or methylene blue can also facilitate UO identification, as long as the kidneys are not obstructed. If available, reviewing computerized tomography urogram, loopogram or cystography results preoperatively can give the surgeon a sense of where to look for the UO during the case.

Even after identification, UO cannulation can still prove difficult in diverted patients due to several factors. One such factor is the ureter’s angle of entry into the diversion, which in some patients can be acute, causing the wire to buckle out of the UO instead of going up the ureter. Actions to improve cystoscope and nephroscope maneuverability within the diversion, as stated above, can improve the angle of approach to the UO and facilitate its cannulation with a wire. Using an angled wire and\or catheter is often essential for cannulation. The choice of wire is also important, as passing a catheter or an instrument over the wire can cause it to buckle inside the diversion and out of the ureter. For that reason, using a stiff hydrophilic wire is advised. Another obstacle to successful UO cannulation is the possible presence of an anastomotic or afferent limb stenosis. This complication is not uncommon among certain diverted patients, and can complicate cannulation further, requiring stricture dilatation before further upper tract access can be achieved.

The last obstacle for upper tract access in diverted patients is ureteral navigation. Over time, chronic reflux or mild obstruction can result in a convoluted ureteral course with many twists and turns. These can prevent the passage of a wire all the way up to the kidney, with the wire getting stuck in one of the ureteral turns. One way to address this issue is to advance an open-ended ureteral catheter over the wire to the level of difficulty and to use a stiff hydrophilic coated wire to navigate and straighten the twists and turns of the ureter up to the kidney. The last maneuver to help negotiate a convoluted ureter is advancing a flexible ureteroscope over the wire to its tip and trying to navigate the ureter all the way to the kidney under direct vision and with fluoroscopic assistance. Once wire or instrumental access to the kidney is achieved, passing a stiff wire to the kidney will many times straighten the ureter and allow for easier navigation and even access sheath placement.

When retrograde access proves impossible, percutaneous antegrade access to the kidney can be attempted. This will not only allow access to the renal collection system, but will also enable antegrade wire passage down the ureter and facilitate further retrograde access. While in theory the antegrade approach negates all the obstacles to retrograde access mentioned above, one needs to remember that retrograde access to the upper tract is usually a prerequisite to percutaneous renal access as it allows the surgeon to distend and opacify the renal collecting system, helping with access acquisition. In the absence of retrograde access, several techniques can be used to assist in antegrade percutaneous access. First, ultrasound (US) can be used to pass a needle into the renal collecting system. Once a needle is in place, the surgeon can use it to distend and opacify the collecting system and either establish access if the original needle is well placed or place a second needle in a more favorable position using US or fluoroscopic guidance. If there is no hydronephrosis and US access proves difficult, intravenous contrast can be given to opacify the renal collecting system and allow for fluoroscopic access to the kidney. A third option is to have interventional radiology establish renal access before surgery.

As with all patients after endoscopic upper tract access, the decision on whether to place a stent or a nephrostomy tube at the end of the procedure depends on the procedure performed, the patient’s anatomy and the perceived irritation of the ureters and kidneys at the end of the procedure. When endoscopic access is relatively straight forward, the ureters are dilated, and the procedure did not require any active dilatation or repeated maneuvering through the ureter, the patient can be left without any ureteral stent or nephrostomy tubes. If access was difficult and there is significant inflammation of the ureter or kidneys, we recommend leaving a ureteral stent or nephrostomy tube for 5–7 days. If stricture dilatation is required, then a ureteral stent for 4–6 weeks is usually suggested.

## Conclusion

While the advantages of endoscopic treatment in diverted patients are obvious, it is far from straightforward. Surgeons attempting this approach should familiarize themselves with the patient’s unique anatomy and have equipment ready to tackle expected challenges. Patients should also be advised that initial attempt at endoscopic access might fail, and that a secondary attempt, sometimes with presurgical access acquisition by interventional radiology, might be required.

## Data availability statement

The original contributions presented in the study are included in the article/supplementary material. Further inquiries can be directed to the corresponding author.

## Author contributions

JM: Writing – original draft. YB: Writing – review & editing. PP: Writing – review & editing. KS: Writing – review & editing. MD: Conceptualization, Writing – review & editing.
